# Prediction of repeated intravenous immunoglobulin resistance in children with Kawasaki disease

**DOI:** 10.1186/s12887-021-02876-w

**Published:** 2021-09-16

**Authors:** Yaheng Lu, Tingting Chen, Yizhou Wen, Feifei Si, Xindan Wu, Yanfeng Yang

**Affiliations:** Department of Pediatric Cardiology, Chengdu Women’s and Children’s Central Hospital, School of Medicine, University of Electronic Science and Technology of China, 611731 Chengdu, China

**Keywords:** Kawasaki disease, Repeated intravenous immunoglobulin resistance, Prediction

## Abstract

**Background:**

Repeated intravenous immunoglobulin (IVIG) resistance prediction is one of the pivotal topics in Kawasaki disease (KD). Those non-responders of repeated IVIG treatment might be improved by an early-intensified therapy to reduce coronary artery lesion and medical costs. This study investigated predictors of resistance to repeated IVIG treatment in KD.

**Methods:**

A total of 94 children with IVIG-resistant KD treated at our hospital between January 2016 and August 2020 were retrospectively analyzed. According to the therapeutic effect of a second dose IVIG treatment, the children were divided into repeated IVIG-responsive group and repeated IVIG-resistant group, and the clinical and laboratory data were compared. Predictors of repeated IVIG resistance and the optimal cut-off value were determined by multiple logistic regression analysis and receiver operating characteristic (ROC) curve analysis.

**Results:**

The Pre-IVIG laboratory data showed the percentage of neutrophils (N%) and levels of serum procalcitonin (PCT), N-terminal pro-brain natriuretic peptide (NT-proBNP) were significantly higher in repeated IVIG-resistant group compared with repeated IVIG-responsive group, while levels of serum sodium and albumin (ALB) were significantly lower (*P* < 0.05). The post-IVIG laboratory values of N% and C-reactive protein (CRP) were significantly higher in the repeated IVIG-resistant group compared with repeated IVIG-responsive group, while hemoglobin and ALB were lower (*P* < 0.05). Pre-IVIG PCT and post-IVIG CRP exhibited AUC of 0.751 and 0.778 respectively in predicting repeated IVIG resistance in KD. Pre-IVIG PCT > 1.81ng/ml (OR 4.1, 95 % CI 1.4 ~ 12.0, *P* < 0.05) and post-IVIG CRP > 45 mg/L (OR 4.6, 95 % CI 1.3 ~ 16.2, *P* < 0.05) were independent predictors of repeated IVIG resistance in KD.

**Conclusions:**

Our study illustrates the serum PCT level before initial IVIG treatment and CRP after initial IVIG could be used to predict repeated IVIG resistance in KD.

## Introduction

Kawasaki disease (KD) is an acute febrile systemic vasculitis syndrome and intravenous immunoglobulin (IVIG) combined with aspirin is the standardized regimen [1]. Approximately 10-20 % of the KD children have a recrudescence or persistent fever at least 36 h following completion of the first dose of IVIG, which is called IVIG-resistant [2]. Japanese risk-scoring systems are used for predicting IVIG-resistance, but it seems irreproducible outside Japan, and attempts to develop similar algorithms have been unsuccessful in Chinese populations [3]. As yet, treatment of resistant KD remains a challenge that needs to be solved [4].

The mechanism of IVIG resistance is not entirely clear now and maybe related to the putative dose-response effect of immunoglobulin [5]. A second dose of IVIG at 2 g/kg is suggested as the first choice for resistant KD in current American Heart Association (AHA) guidelines [1]. However, approximately 10 % of KD children might develop to both initial and repeated IVIG resistance [6], and often require additional interventions, such as corticosteroid, infliximab, plasma exchange and cytotoxic agents [7]. An intensified initial rescue therapy may reduce the occurrence of repeated IVIG resistance, but whether apply to every resistant KD child is controversial when taking into account potential adverse outcomes and the economic cost of the treatment [8–10]. It seems more reasonable to consider intensified therapy if KD children with repeated IVIG resistance,that have high risk of coronary artery aneurysm (CAA) [11]. Therefore, early identification of repeated IVIG resistance could help physicians make smarter therapeutic decisions to reduce CAA and medical costs. Herein, we performed this study to explore predictors of repeated IVIG resistance.

## Materials and methods

### Study subjects

**Fig. 1:**
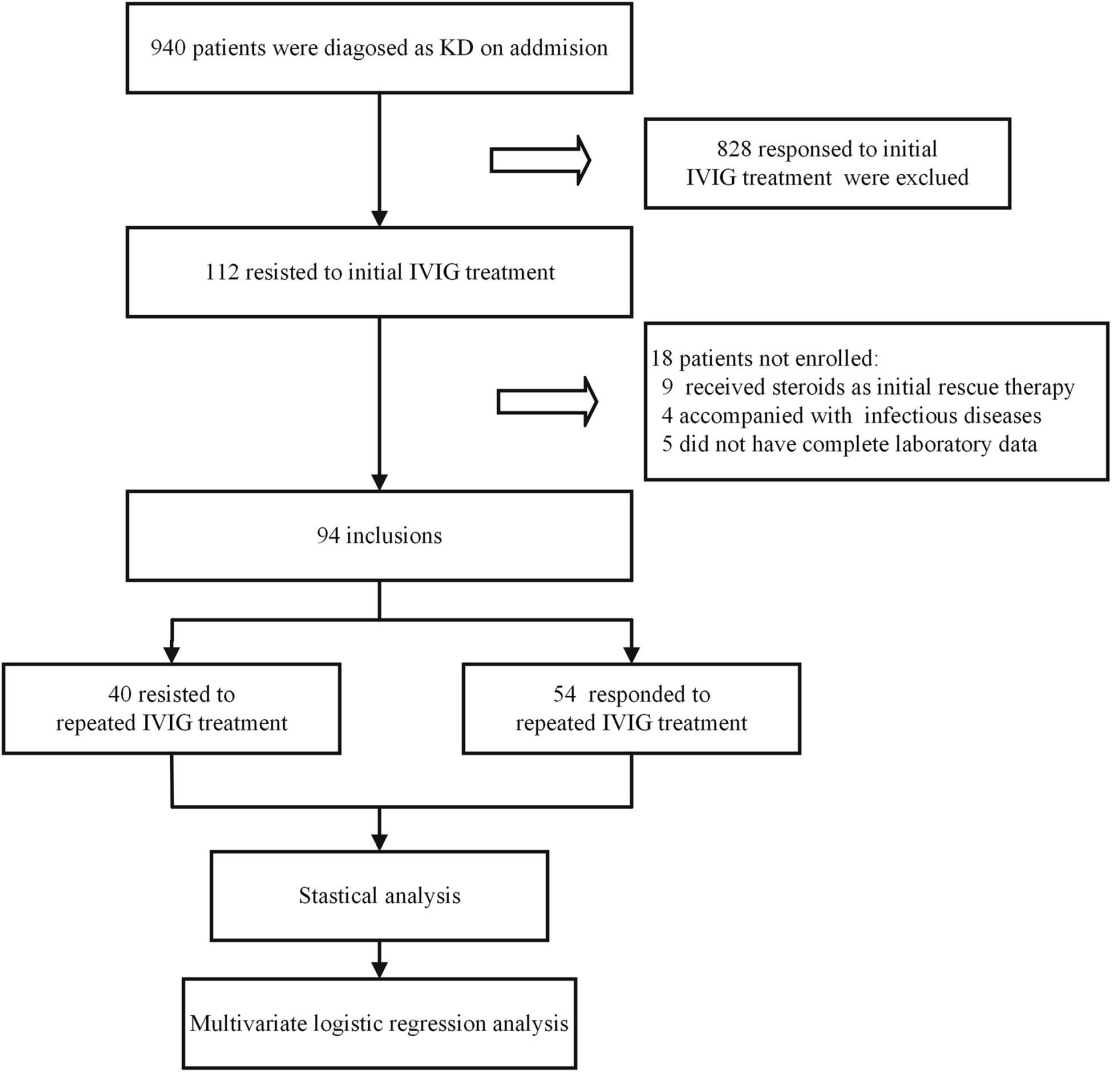
The flowchart of our retrospective study

From January 2016 to August 2020, 940 children with KD were admitted and managed at Chengdu Women’s and Children’s Central Hospital, School of Medicine, University of Electronic Science and Technology of China (UESTC). The diagnosis of complete and incomplete KD was established according to the American Heart Association guideline in 2004 [12]. Among them, 112 KD children developed resistance to initial standard IVIG (2 g/kg/d for 1 day) therapy. Initial IVIG resistance was defined as recurrent or persistent fever (under-arm temperature ≥ 37.5℃) for at least 36 h but not longer than 7 days after initial IVIG treatment. Of the 112 initial IVIG-resistant KD children, 9 patients that received steroids as initial rescue therapy instead of a second dose IVIG treatment were excluded, and another 9 children were excluded because of incomplete laboratory data (*n* = 5) or accompanying infectious diseases (*n* = 4). Finally, 94 initial IVIG-resistant KD children were included in the study (Fig. 1).

For 94 KD children with initial IVIG resistance, a second dose IVIG (2 g/kg/d for 1 day) was administered according to expert consensus on the diagnosis and treatment of KD in China, and 40 of them developed repeated IVIG resistance. Repeated IVIG resistance was defined as recurrent or persistent fever (under-arm temperature ≥ 37.5℃) for at least 36 h after the second IVIG administration. At last, the initial IVIG-resistant KD children were divided into repeated IVIG-responsive group (*n* = 54) and repeated IVIG-resistant group (*n* = 40).

This retrospective study was approved by the Ethics Committee of Chengdu Women’s and Children’s Central Hospital, School of Medicine, UESTC, and with the 1964 Helsinki declaration and its later amendments or comparable ethical standards. The requirement for informed patient consent was waived.

### Data collection

Clinical and laboratory data were collected through medical record review. Clinical data such as age, sex, diagnosis of incomplete KD, time of first and second dose IVIG were collected. Laboratory data on admission pre-IVIG treatment were collected, including white blood cell count (WBC), platelet (PLT), hemoglobin (Hb), percentage of neutrophils (N%), C-reactive protein (CRP), total bilirubin (TB), serum albumin (ALB), erythrocyte sedimentation rate (ESR), serum alanine aminotransferase (ALT), serum aspartate transaminase (AST), serum sodium (Na+), serum creatinine, blood urea nitrogen, activated partial thromboplastin time (APTT), prothrombin time (PT), D-dimer, procalcitonin (PCT), N-terminal pro-brain natriuretic peptide (NT-proBNP). Laboratory data post-IVIG treatment before the second dose of IVIG including WBC, PLT, Hb, N%, CRP, ALB, ALT and AST were collected.

The value of coronary artery diameter measured by echocardiography before initial IVIG treatment and at discharge was selected and used to calculate the z score using the formula by Dallaire and Dahdah [13]. As there is no normal value of coronary artery diameter in Chinese children, CAA was defined as *Z* score of ≥ 2.5 in at least one of the following coronary arteries: right, left anterior descending, and left main according to the AHA guideline in 2004 [14].

### Statistical analysis

All statistical analyses were performed using IBM SPSS Statistics ver. 21.0 (IBM Co., Armonk, NY, USA). All continuous variables are described as median with interquartile range (25–75 % percentile). All categorical variables are described as a frequency with percentage. A χ2 test was performed to compare categorical variables. Student’s t-test was used for normally distributed continuous variables. The Mann-Whitney U-test was used when the distribution was skewed. Receiver operating characteristic curve (ROC) analysis for the predictor was performed, the sensitivity and specificity were calculated, and the cutoff value was determined by the Youden index. Multivariate logistic regression analyses were performed to determine predictors of repeated IVIG resistance. Statistical significance was defined as a *P* < 0.05.

## Results

Table 1 exhibits clinical data of the repeated IVIG-responsive group and repeated IVIG-resistant group. The clinical variables of sex, age, the proportion of incomplete KD, the incidence of CAA, and the time of IVIG treatment showed no significant differences between the two groups (*P* > 0.05).

**Table 1:** Clinical data of the study subjects

Parameter	repeated IVIG-responsive group (*n* = 54)	repeated IVIG-resistant group (*n* = 40)	*p*
Age(months)	35.0(13.0 ~ 60.0)	38.5(19.0 ~ 50.7)	0.807
Boys (%)	44(81.4 %)	26(65.0 %)	0.070
iKD[(%)]	4(7.4 %)	4(10.0 %)	0.656
Time of first IVIG (day)	6.0(5.0 ~ 6.0)	5.5(5.0 ~ 6.0)	0.535
Time of second IVIG (day)	10.0(8. 0 ~ 12.0)	9.00(8.0 ~ 12.7)	0.307
NO. of CAA before initial IVIG (%)	12(22.2 %)	12(30.0 %)	0.393
NO. of CAA at discharge (%)	12(22.2 %)	14(35.0 %)	0.171

*Statistically significant (*P* < 0.05)

*IVIG* intravenous immunoglobulin, *iKD* incomplete kawasaki disease, *CAA* coronary artery aneurysm, *NO.* number

**Fig. 2:**
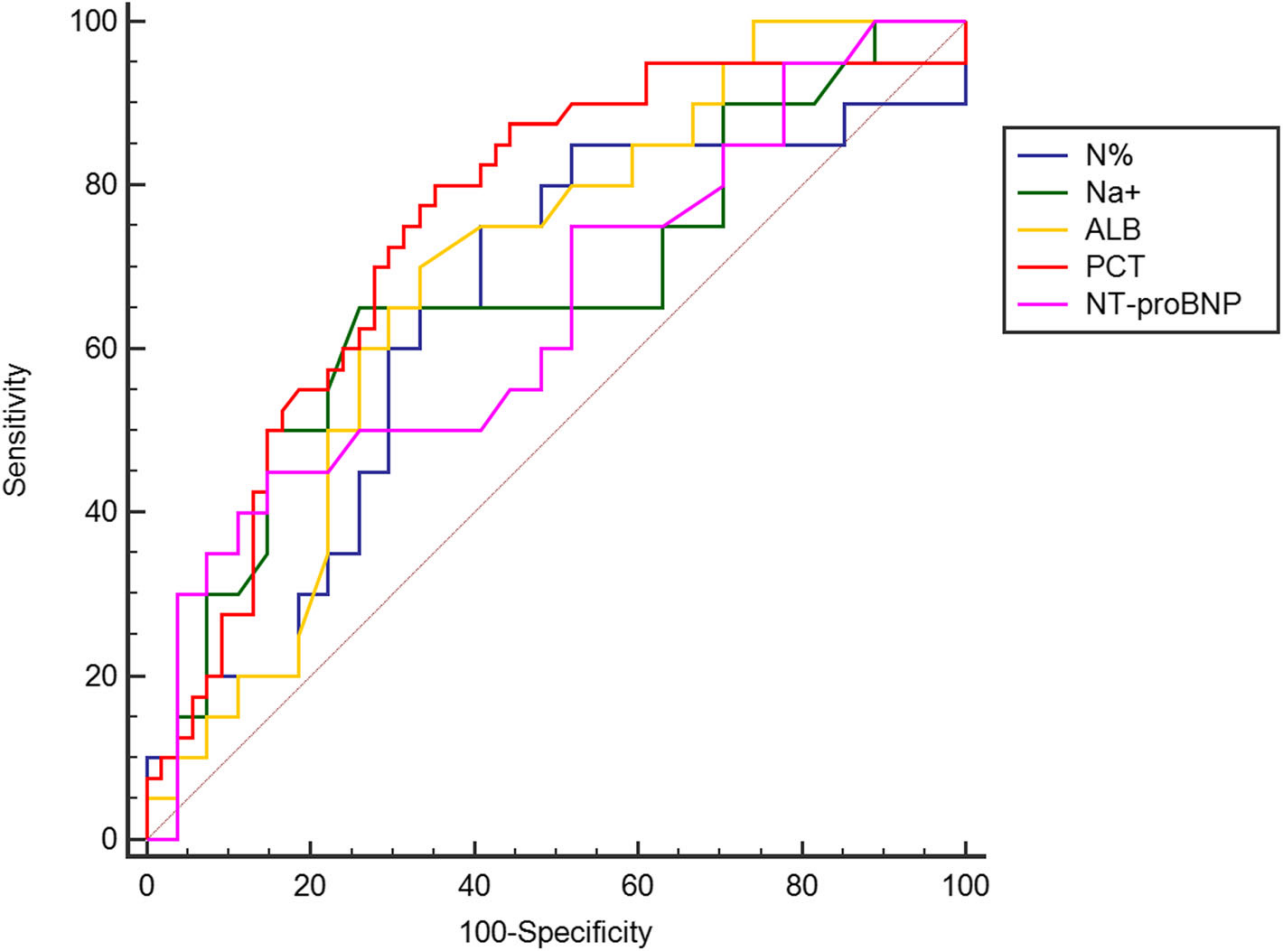
ROC curve analysis of pre-IVIG laboratory data in predicting repeated IVIG resistance N%: percentage of neutrophils; Na+: serum sodium; ALB: albumin; PCT: procalcitonin; NT-proBNP: N-terminal pro-brain natriuretic peptide; AUC, area under curve

Table 2 exhibits pre-IVIG laboratory data. N% and levels of serum PCT, NT-proBNP were significantly higher in the repeated IVIG-resistant group compared with repeated IVIG-responsive group, while levels of serum Na + and ALB were significantly lower (*P* < 0.05). The ROC curves using pre-IVIG laboratory data of N%, Na+, ALB, PCT and NT-proBNP to predict repeated IVIG resistance were analyzed (Fig. 2; Table 3). Pre-IVIG PCT exhibited the largest AUC (0.751) compared with other indicators. According to the maximum Youden index, the classical cutoff points for PCT, N%, Na+, ALB, and NT-proBNP were 1.81 ng/ml, 133.8mmol/l, 32.2U/L, 965.8pg/dl, respectively. The sensitivity of PCT was revealed to be the highest with a value of 80.0 %, and the specificity of NT-proBNP was determined to be the highest with a value of 85.1 %. Using N%>76.5 %, Na + ≤ 133.8mmol/l, ALB ≤ 32.2ng/ml, PCT > 1.81ng/ml, NT-proBNP > 965.8 pg/ml before initial IVIG treatment as binary independent variable, multivariate logistic regression analysis for repeated IVIG resistance in KD was analyzed (Table 4). The results showed that pre-IVIG PCT > 1.81ng/ml was an independent predictor for repeated IVIG resistance (OR 4.1, 95 % CI 1.4 ~ 12.0, *P* < 0.05).

**Table 2:** Laboratory data of the study subjects pre-IVIG treatment

Parameter	repeated IVIG-responsive group (*n* = 54)	repeated IVIG-resistant group (*n* = 40)	*p*
WBC count (×10 ^9^ /L)	16.51(10.25 ~ 20.59)	15.39(11.07 ~ 17.74)	0.807
N%	73.50(70.50 ~ 86.60)	84.60(74.97 ~ 89.15)	0.017*
Hb (g/L)	110(96 ~ 121)	103(92 ~ 115)	0.061
PLT (×10 ^9^ /L)	284(215 ~ 381)	264(190 ~ 339)	0.173
CRP (mg/L)	106.5(73.0 ~ 141.0)	114.0(83.8 ~ 166.5)	0.119
ESR(mm/h)	81.0(52.0 ~ 100.0)	86.5(65.8 ~ 119.5)	0.338
ALT (U/L)	61.4(17.0 ~ 153.6)	66.0(28.0 ~ 136.9)	0.482
AST (U/L)	67.1(35.9 ~ 133.9)	41.5(33.2 ~ 97.7)	0.178
Na+ (mmol/L)	135.0(133.8 ~ 137.8)	133.0(130.3 ~ 136.9)	0.006*
ALB (g/L)	33.5(30.2 ~ 37.1)	30.2(29.3 ~ 33.3)	0.001*
TB (umol/L)	12.6(9.8 ~ 17.8)	14.9(11.4 ~ 18.9)	0.167
Creatinine (umol/L)	30.8(26.7 ~ 35.0)	32(29.0 ~ 35.8)	0.234
Urea nitrogen (mmol/L)	3.19(2.79 ~ 3.49)	3.33(2.83 ~ 3.90)	0.063
APTT(s)	47.8(43.5 ~ 52.5)	44.6(41.2 ~ 52.5)	0.351
PT(s)	15.3(14.5 ~ 16.8)	16.0(15.4 ~ 16.7)	0.052
D-dimer (ug/ml)	1.92(1.36 ~ 3.88)	2.34(1.44 ~ 3.89)	0.204
PCT (ng/ml)	1.40(0.99 ~ 2.84)	3.81(1.90 ~ 10.51)	< 0.001*
NT-proBNP(pg/ml)	487.1(182.3 ~ 763.9)	669.8(303.5 ~ 1493.8)	0.015*

*Statistically significant (*P* < 0.05)

*WBC* white blood cell, *Hb* hemoglobin, *PLT* platelet, *N%* percentage of neutrophils, *CRP* C-reactive protein, *ESR* erythrocyte sedimentation rate, *ALT* alanine aminotransferase, *AST* aspartate transaminase, *Na+* serum sodium, *ALB* albumin, *TB* total bilirubin, *APTT* activated partial thromboplastin time, *PT* prothrombin time, *PCT* procalcitonin, *NT-proBNP* N-terminal pro-brain natriuretic peptide

**Table 3:** ROC curve analysis of pre-IVIG laboratory data to predict repeated IVIG resistance

Indicator	AUC	*P*-value	95 % CI	Cut-off	Sensitivity (%)	Specificity (%)	Youden index
N%	0.644	0.016	0.539 ~ 0.741	76.5 ^a^	75.00	59.26	0.3426
Na+	0.667	0.005	0.562 ~ 0.761	133.8 ^b^	65.00	74.07	0.3907
ALB	0.690	< 0.001	0.586 ~ 0.781	32.2 ^c^	70.00	66.67	0.3667
PCT	0.751	< 0.001	0.710 ~ 0.879	1.81 ^d^	80.00	64.80	0.4481
NT-proBNP	0.647	0.012	0.542 ~ 0.743	965.8 ^e^	45.00	85.19	0.3019

^a^ Values are %; ^b^ Values are mmol/l; ^c^ Values are U/L ; ^d^ Values are ng/ml; ^e^ Values are pg/ml

*N%* percentage of neutrophils, *Na+* serum sodium, *ALB* albumin, *PCT* procalcitonin, *NT-proBNP* N-terminal pro-brain natriuretic peptide, *AUC* area under curve

**Table 4:** Multivariate logistic regression model composing pre-IVIG laboratory data for predicting repeated IVIG resistance

Indicator	*P*	*OR*	*95 %CI*
N%>76.5 %	0.179	2.183	0.699 ~ 6.815
Na + ≤ 133.8mmol/l	0.421	1.611	0.504 ~ 5.153
ALB ≤ 32.2ng/ml	0.146	2.226	0.758 ~ 6.541
PCT > 1.81ng/ml	0.008	4.161	1.441 ~ 12.017
NT-proBNP > 965.8 pg/ml	0.177	2.281	0.690 ~ 7.541

*N%* percentage of neutrophils, *Na+* serum sodium, *ALB* albumin, *PCT* procalcitonin, *NT-proBNP* N-terminal pro-brain natriuretic peptide, *AUC* area under curve

**Fig. 3:**
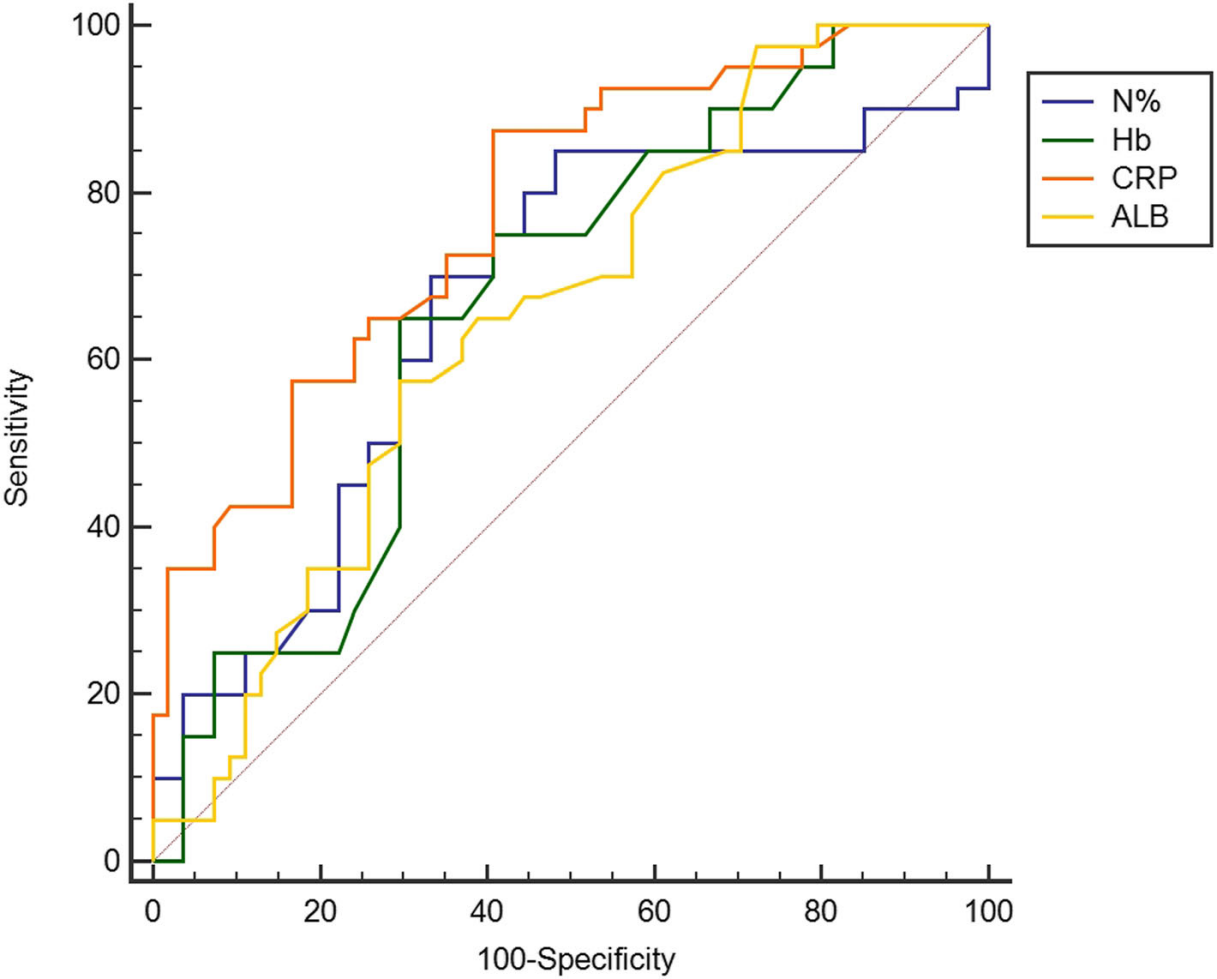
ROC curve analysis of post-IVIG laboratory data in predicting repeated IVIG resistance N%: percentage of neutrophils; Hb: hemoglobin; CRP: C-reactive protein; ALB: albumin

Table 5 exhibits post-IVIG laboratory data. N% and CRP were significantly higher in the repeated IVIG-resistant group compared with repeated IVIG-responsive group, while Hb and levels of serum ALB were significantly lower (*P* < 0.05). The ROC curves using post-IVIG laboratory data of N%, Hb, CRP, and ALB to predict repeated IVIG resistance were analyzed (Fig. 3; Table 6). Post-IVIG CRP exhibited the largest AUC (0.778) compared with other indicators. The classical cutoff points for N%, Hb, CRP, and ALB post IVIG were 62.5 %, 97 g/L, 45.0 mg/L, 29.7U/L, respectively. The sensitivity of CRP was revealed to be the highest with a value of 87.50 %. Using N%>62.5 %, Hb ≤ 97 g/L, CRP > 45 mg/L, ALB ≤ 29.7U/L post initial IVIG treatment as binary independent variable, multivariate logistic regression analysis for repeated IVIG resistance in KD was analyzed (Table 7). The results showed that post-IVIG CRP > 45 mg/L was an independent predictor (OR 4.6, 95 % CI 1.3 ~ 16.2, *P* < 0.05).

**Table 5:** Laboratory data of the study subjects post-IVIG treatment

Parameter	repeated IVIG-responsive group (*n* = 54)	repeated IVIG-resistant group (*n* = 40)	*p*
WBC count (×10 ^9^ /L)	14.26(12.93 ~ 15.66)	14.03(9.96 ~ 16.19)	0.125
N%	62.50(59.50 ~ 75.60)	74.40(64.77 ~ 78.95)	0.008*
Hb (g/L)	105(93 ~ 116)	96(85 ~ 107)	0.004*
PLT (×10 ^9^ /L)	370(295 ~ 461)	354(280 ~ 429)	0.253
CRP (mg/L)	41.0(27.3 ~ 65.8)	76.0(50.0 ~ 124.0)	< 0.001*
ALT (U/L)	46.4(14.6 ~ 138.6)	56.0(23.0 ~ 122.4)	0.763
AST (U/L)	57.1(28.7 ~ 123.9)	33.0(25.1 ~ 89.7)	0.081
ALB (g/L)	31.2(28.6 ~ 33.1)	29.4(27.6 ~ 31.5)	0.010*

*Statistically significant (*P* < 0.05)

*WBC* white blood cell, *N%*: percentage of neutrophils, *Hb* hemoglobin, *PLT* platelet, *CRP* C-reactive protein, *ALT* alanine aminotransferase, *AST* aspartate transaminase, *ALB* albumin

**Table 6:** ROC curve analysis of post-IVIG laboratory data to predict repeated IVIG resistance

Indicator	AUC	*P*-value	95 % CI	Cut-off	Sensitivity (%)	Specificity (%)	Youden index
N%	0.665	0.005	0.560 ~ 0.759	62.5 ^a^	85.00	51.85	0.3685
Hb	0.667	0.003	0.562 ~ 0.761	97^b^	65.00	70.37	0.3537
CRP	0.778	< 0.001	0.680 ~ 0.857	45.0 ^c^	87.50	59.26	0.4676
ALB	0.652	0.007	0.547 ~ 0.747	29.7 ^d^	57.50	70.37	0.2787

^a^ Values are %; ^b^ Values are g/L; ^c^ Values are mg/L ; ^d^ Values are U/L

*N%* percentage of neutrophils, *Hb* hemoglobin, *CRP* C-reactive protein, *ALB* albumin

**Table 7:** Multivariate logistic regression model composing post-IVIG laboratory data for predicting repeated IVIG resistance

Indicator	*P*	*OR*	*95 %CI*
N%>62.5 %	0.173	2.290	0.696 ~ 7.538
Hb ≤ 97 g/L	0.205	1.949	0.694 ~ 5.476
CRP > 45 mg/L	0.016	4.664	1.339 ~ 16.247
ALB ≤ 29.7U/L	0.174	1.973	0.740 ~ 5.256

*N%* percentage of neutrophils, *Hb* hemoglobin, *CRP* C-reactive protein, *ALB* albumin

## Discussion

There have been numerous studies of initial IVIG resistance in KD, but few reports on repeated IVIG resistance. In our retrospective investigation, the incidence of initial IVIG resistance was about 11.9 % (112/940), and the rate of repeated IVIG resistance was about 4.2 % (40/940). It has been reported that age less than 3 months, incomplete KD, incidence of CAA and initial administration of IVIG ≤ 4.0 days were associated with initial IVIG resistance [15, 16]. But, these clinical data were not associated with repeated IVIG resistance in our research. The hypercoagulation of increased APTT, PT and D-dimer and the abnormal liver function of increased ALT, AST, TB were confirmed as the risk factors favor initial IVIG resistance [17, 18]. These laboratory data failed to be the predictor of repeated IVIG resistance in our study, which was not found in other studies either [18, 19].

However, pre-IVIG laboratory data showed that KD children with repeated IVIG resistance had higher N%, PCT, and lower ALB, Na+ compared with KD children responding to repeated IVIG treatment, which is also confirmed in other studies [12, 17, 20], indicating more serious inflammation and increased vascular permeability after KD onset in these children [21]. A significantly higher level of NT-proBNP, which is an important cardiac biomarker that associating with ventricular myocyte ischemia and hypoxia [22], was observed in these children, indicating more serious myocardial injury. Among the post-IVIG laboratory data, values of N% and CRP were higher in the repeated IVIG-resistant group, while Hb and ALB were lower, indicating more inadequate remission of systemic inflammation and vasculitis in children with repeated IVIG resistance after initial IVIG treatment, that may require more aggressive rescue therapy than IVIG alone.

Predictive values of pre- and post-IVIG laboratory data on repeated IVIG resistance were further explored respectively. The pre-IVIG regression model showed PCT > 1.81ng/ml before initial IVIG treatment was an independent predictor of repeated IVIG resistance in KD. Comparing with CRP as well as the other inflammation indicator, PCT may reflect uncontrollable hypercytokinemia faster along with the involvement of TNF-α signaling pathway in KD, based on the characteristics of early elevation and shorter half time [23]. Pre-IVIG NT-proBNP yielded the highest specificity, but it failed to predict repeated IVIG resistance, the reason may be due to relatively low sensitivity of 45.00 %. At its root, perhaps, the mechanism of IVIG resistance is an immune response that causes systemic vasculitis, not only damage to the coronary arterial wall [24]. The increase of PCT in monocytes and granulocytes mediated by the activated innate immune system after KD onset through tumor necrosis factor (TNF) signaling pathways could lead to impairing of endothelial cell [25, 26], which may relate to the etiology of Kawasaki disease. Though CRP has been known to reflect the extent of systemic inflammation and vasculitis, it alone does not always reflect the severity of KD or the treatment response of IVIG [20]. CRP > 45 mg/L after initial IVIG exhibited predictive value of repeated IVIG resistance in post-IVIG regression model. Persistently elevated CRP after initial IVIG may suggest a poorly responding severe form of KD, that was found in other studies also [27].

At present, whether enhancing anti-inflammatory treatment to reduce IVIG-resistance could decrease the incidence of CAA remains controversial [28]. In our study, the incidence of CAA during hospitalization was relatively higher in KD children with repeated IVIG resistance, indicating the potential benefit. Pulse intravenous methylprednisolone (PSL) (10–30 mg/kg/day for three consecutive days) followed by oral prednisone (2 mg/kg/day) tapered over seven days was given to KD children with repeated IVIG resistance in our study, and all of them were relieved effectively. No patients received any additional treatment such as infliximab, plasma exchange, or cyclosporine. Recent studies demonstrated the IVIG plus PSL regimen as initial therapy for severe KD or rescue therapy following resistance to initial IVIG was more effective in terms of achieving defervescence than IVIG alone [29, 30]. Based on the predictor in our research, IVIG plus PSL as initial therapy for KD children with Pre-IVIG PCT > 1.81ng/ml and as initial rescue therapy for KD children with post-IVIG CRP > 45 mg/L may be a reasonable intensified treatment strategy to reduce initial or repeated IVIG resistance respectively.

This study has some limitations. First, the sample size of this study is small, further multicenter prospective studies are needed to confirm our findings. Second, the present study had strict inclusion and exclusion criteria. The findings in our study were only applicable to KD patients receiving standardized IVIG treatment. Third, the parameter of post-IVIG laboratory data in our study was limited, more indicators need to be included prospectively. Fourth, every subject in this study was Chinese. Therefore, our findings should be examined in other ethnic groups.

## Conclusions

KD children with repeated IVIG resistance have more serious inflammation and myocardial injury compared with KD children responding to repeated IVIG treatment. KD patients with PCT > 1.81ng/ml before initial IVIG treatment and CRP > 45 mg/L after initial IVIG might be at higher risk of developing repeated IVIG resistance, which may require an early-intensified therapy.

## Data Availability

The datasets used and analyzed during the current study are not publicly available due to limitations of ethical approval involving the patient data and anonymity but are available from the corresponding author on reasonable request.

## Abbreviations

KD: Kawasaki disease
IVIG: Intravenous immunoglobulin
CAA: Coronary artery aneurysm
N%: Percentage of neutrophils
ALB: Albumin
Na+: Serum sodium
PCT: Procalcitonin
NT-proBNP: N-terminal pro-brain natriuretic peptide
Hb: hemoglobin
CRP: C-reactive protein
